# Raman Spectroscopy as Spectral Tool for Assessing the Degree of Conversion after Curing of Two Resin-Based Materials Used in Restorative Dentistry

**DOI:** 10.3390/diagnostics12081993

**Published:** 2022-08-17

**Authors:** Eduard Gatin, Stefan-Marian Iordache, Elena Matei, Catalin-Romeo Luculescu, Ana-Maria Iordache, Cristiana Eugenia Ana Grigorescu, Roxana Romanita Ilici

**Affiliations:** 1 Faculty of Medicine, University of Medicine ‘‘Carol Davila’’, Blv. Eroii Sanitari 8, Sector 5, 020021 Bucharest, Romania; 2 Faculty of Physics, University of Bucharest, Atomistilor 405, 077125 Magurele, Romania; 3 Optospintronics Department, National Institute for Research and Development for Optoelectronics—INOE 2000, 077125 Magurele, Romania; 4 National Institute of Materials Physics, Atomistilor 405A, 077125 Magurele, Romania; 5 National Institute for Laser, Plasma and Radiation Physics, CETAL, 077125 Magurele, Romania; 6 Faculty of Dental Medicine, University of Medicine “Carol Davila”, Plevnei Route No. 17-23, Sector 1, 020021 Bucharest, Romania

**Keywords:** raman spectrometry, conversion rate, resin-based composite, dentistry restoration

## Abstract

(1) Background: The treatment of dental cavities and restoration of tooth shape requires specialized materials with specific clinical properties, including being easy to model, light-cured, having a natural color, reduced shrinkage, a hardness similar to hydroxyapatite, and no leakage. The dimensional stability of resin composite materials is affected by polymerization shrinkage, degree of conversion (number of π carbon bonds converted into σ ones), thermal contraction and expansion, and interactions with an aqueous environment. (2) Methods: The materials used in our investigation were two composite resins with similar polymer matrices, but different filler (micro/nano filler). To evaluate the properties of samples, we employed the pycnometer technique (pycnometer from Paul Marienfeld Gmbh, Lauda-Königshofen, Germany), RAMAN spectroscopy technique (MiniRam Equipment from B&W Tek Inc., Plainsboro Township, NJ, USA; 785 nm laser source), SEM and EDX (FEI Inspect S.). (3) Results: The size of the filler plays an important role in the polymerization: for the pycnometric results, the larger particle filler (Sample 1) seems to undergo a rapid polymerization during the 45 s curing, while the nanoparticle filer (Sample 2) needs additional curing time to fully polymerize. This is related to a much larger porosity, as proved by SEM images. The lower degree of conversion, as obtained by Raman spectroscopy, in the same geometry means that the same volume is probed for both samples, but Sample 1 is more porous, which means less amount of polymer is probed for Sample 1. (4) Conclusions: For the two composites, we obtained a degree of conversion of 59% for Sample 1 and 93% for Sample 2, after 45 s of curing.

## 1. Introduction

The treatment of dental cavities and restoration of tooth shape requires specialized materials with specific clinical properties, including being easy to model, light-cured, having a natural color, reduced shrinkage, a hardness similar to hydroxyapatite, and no leakage [1]. A single unique material does not possess all these properties but by combining an inorganic filler with a polymer matrix, one obtains a resin-based material that has all of them. Thus, since 1958, resin-based composites have been increasingly used as dental restorative materials or dental cements, because of their good esthetic and mechanical properties [2]. The first generation of composites introduced in the clinical practice was in the 1980s with Bis-GMA as a principal component, followed in the 1990s with the second generation of resin composites based on polyglass materials. First-generation composite resin materials are limited by three drawbacks: (1) unreacted monomer left after polymerization; (2) less than optimal hardness which limits the application, e.g., the material cannot be used for all teeth, only for the ones that exhibit lower masticatory loads during chewing; and (3) fatigue and cracking of the polymer due to shrinkage after polymerization [3,4]. Among all, the most important factor is polymerization shrinkage [5] that causes marginal gaps between the enamel and resin and can lead to premature failure of the restoration [2]. Debonding may create plastic deformation, marginal leakage and staining, postoperative sensitivity, and increase the risk of secondary caries formation and pulpal inflammation. Polymerization shrinkage will impact the adhesion of the polymer–tooth interface, which is an inevitable effect of the curing process as monomer molecules, converted into a crosslinked polymer network, exchange van der Waals bonds for shorter covalent bonds [6,7,8]. This volumetric shrinkage causes stress in confined environments such as tooth cavities [9,10].

Incorporation of filler into resin matrix greatly influences and improves material properties, provided that filler particles are bonded to polymer matrix or otherwise it may actually weaken the resin. Thus, filler–polymer matrix coupling determines, to a large extent, the mechanical strength and clinical longevity of dental composites. Several types of filler are commonly used in clinical practice, including glass, pyrogenic silica, zirconia, organic filler [11], metal/alloy [12], and, lately, bioactive glass [13,14].

Despite the variety of formulations, the polymerization shrinkage following curing of the resin presents a major problem. The longevity of the composite resin material is determined by the degree of conversion. By analyzing the degree of conversion, one could establish a direct correlation between the quality of polymerization and the hardness of the composite [15]. Spectrometry is the main technique to determine the residual monomers due to its sensitivity towards conjugated structures (e.g., aromatic rings, C=C, etc.).

The objective of this study is to understand the local stresses and their dynamics for different intervals of polymerizations, as well as imaging the tooth–resin interface. We employed RAMAN investigation since it is a proven method to calculate the degree of conversion [16,17]. Our aim is to reveal the differences between two widely used dental materials, commercialized as “nano” and “micro” photopolymerizable dental composites and demonstrate their behavior at the boundary between dentin and enamel. Identification of this boundary could allow dental professionals to choose the appropriate material for the right tooth (for example, teeth used in biting need sturdier materials than teeth used for chewing [18]). We revealed important aspects regarding the chemical specimens in the dentin–composite and enamel–composite interface areas by EDX and mapping methods (secondary electrons, backscattered electrons).

## 2. Materials and Methods

The materials used in our investigation were two composite resins (RBC) with the same polymer matrix (BIS-GMA, TEGDMA, BIS-EMA), but different filler (micro/nano filler) commercially available on the market in Romania. Sample 1 is Valux^TM^Plus Restorative 3M ESPE, a first-generation composite resin material which contains Bis-GMA and TEGDMA resins and 66% vol. zirconia/silica inorganic filler with a particle size range of 3.5 to 0.01 micron. Sample 2 is Brilliant^TM^NG Coltene, a second-generation composite resin material which is a nanocomposite with pre-polymerized particle filling with high nanometric particle content. Sample 1 comes in 5 shades available in 4 g syringes, while Sample 2 has a duo shade system in the same syringe simplifying the selection of color and inventory of syringes [16,17,18]. Both samples have indications for anterior and posterior restorations including inlays, onlays, and veneers. The characteristics of the samples are presented in Table 1.

Small discs with a diameter of 5 mm and a thickness of 1 mm were prepared in order to investigate the density of the material. Curing was performed with an LED lamp (light emitting diodes, λ = 420 ÷ 480 nm, Kerr Corp., Orange, CA, USA), with different curing programs (variable light intensity, different time rates and mixed). As a first step of the investigation, the variation in the density of the samples was examined before and after curing (curing time 45 s) using the Archimedes’ principle (pycnometer from Paul Marienfeld Gmbh, Lauda-Königshofen, Germany). Density correction for water was applied to the results.

For the composite restoration, the standardized mesio-occluso-distal (MOD) cavity preparation was performed in an extracted upper human premolar, using a medium-grained diamond bur. The molar extraction was performed during the complex treatment of the upper jaw, for a sinus lift augmentation of a patient as reported [19]. The same bioethical approval was applied. Sample 1 (Valux^TM^Plus Restorative 3M ESPE) was incrementally applied and light-cured for 20 s from the LED light-curing system, shifting output intensity from 1.100 mW/cm^2^ to a peak of 1.330 mW/cm^2^ multiple times throughout the curing cycle.

Raman spectroscopy evaluation was performed with a BTR111—785 RAMAN spectrometer device (λ = 785 nm, output power *p* = 300 mW, and spectral resolution 4 cm^−1^) in the Raman shift range 100–2200 cm^−1^. The spectrometer is equipped with BAC101 immersion Raman probe, with the minimum spot size for excitation of 100 µm at 0.5 mm. The 785 nm laser power used for all measurements was 30 mW (10% from 300 mW available) which gives an irradiance of about 382 W/cm^2^. The laser spot and irradiance levels were kept constant during Raman spectra acquisition. Energy Dispersive X-Ray Spectroscopy (EDX) and Scanning Electron Microscopy (SEM) were performed in a Zeiss Evo 50 XVP microscope, at room temperature (RT), equipped with a secondary electron detector in low vacuum and a solid state BSE detector, plus an auxiliary micro analytic SDD radiation detector [19,20,21].

## 3. Results and Discussion

The results of the density variation for the two samples are presented in Table 2. According to these values, a variation of more than 20% was observed for both samples, slightly lower for the microcomposite than for the nanocomposite. Variation in density (%) is equivalent to the experimentally determined polymerization shrinkage [22] and calculated with the formula:

(1) $$Variation\;\left(\%\right)=\frac{Density\;before\;curing-Density\;after\;curing}{Density\;before\;curing\;}\times 100$$

Considering that the mass of the composite remains the same and that density = mass/volume, a decrease of 20% indicates an equal expansion of the volume. Although the results suggest that polymerization shrinkage is higher for Sample 2 (1217.557) and lower for Sample 1 (1514.511), by using the volume of a cylinder (V = πr^2^h) and by calculating the ΔV as the difference between V_initial_–V_final_, we obtain a difference of 5.375 mm^3^ for Sample 1 and 4.375 mm^3^ for Sample 2. This means that the volume increase is higher for Sample 1, which could mean a better polymerization (almost all the monomers are polymerized, and the volume is expanded due to polymerization) and lower for Sample 2, with only a 4.375 mm^3^ increase.

Thus, the size of the filler plays an important role in the polymerization: the larger particle filler (Sample 1) undergoes a rapid polymerization during the 45 s curing, while the nanoparticle filer (Sample 2) needs additional curing time to fully polymerize. The lower polymerization shrinkage could suggest an unfinished polymerization process.

### 3.1. SEM Microscopy

**Figure 1:**
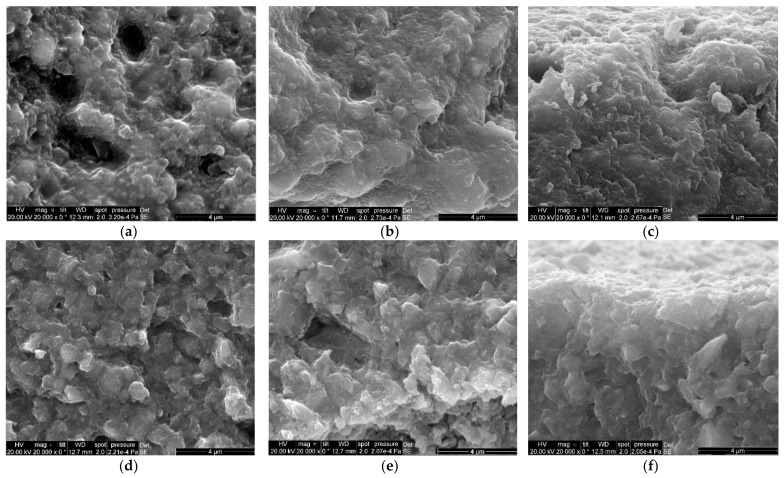
SEM micrographs of the samples: (**a**) Sample 1-the microcomposite, seen in the middle of the section area; (**b**) Sample 1-the side facing the curing lamp; (**c**) Sample 1-the side opposite to the curing lamp; (**d**) Sample 2-the nanocomposite, in the middle of the section area; (**e**) Sample 2-the side facing the curing lamp; (**f**) Sample 2-the side opposite to the curing lamp.

Following density measurements, an imaging investigation was performed using SEM. The samples were sectioned in depth and imaging was performed in the middle of the section, but also on the side facing the curing lamp and the opposite side. The SEM micrographs show that the microcomposite has a porous structure (Figure 1a), this porosity being maintained on both the side facing the curing lamp (Figure 1b) and the opposite side (Figure 1c). The nanocomposite shows a more compact surface, with rare and fine pores (Figure 1d). However, there are large holes on the side facing the curing lamp (Figure 1e), while the compactness of the back side surface is evident (few gaps in material—Figure 1f).

### 3.2. EDX Investigations

**Figure 2:**
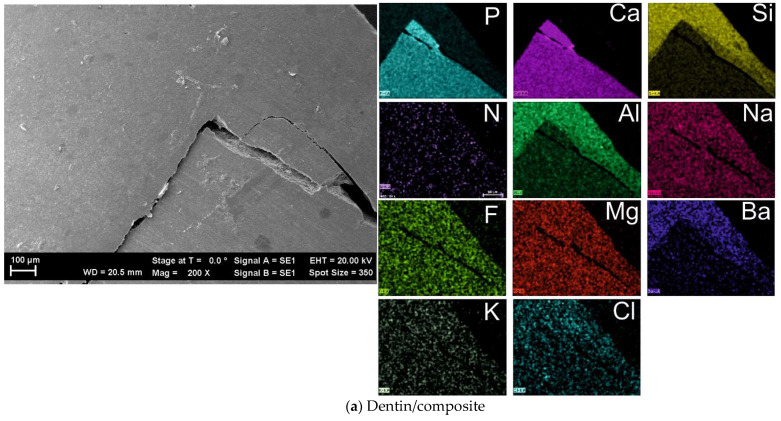

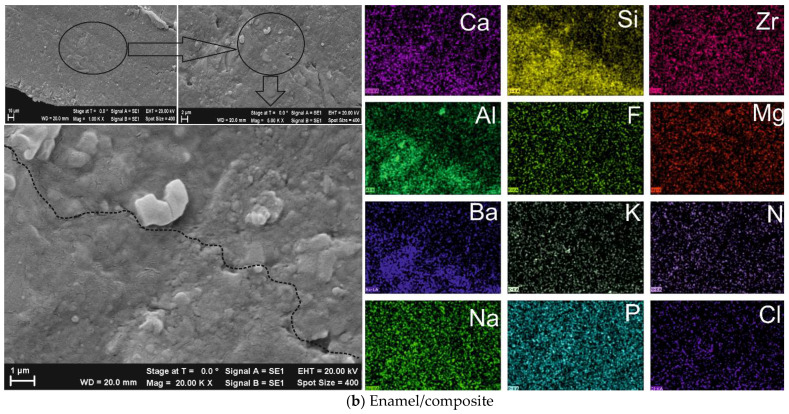
EDX measurements of the dentin/composite border (**a**) and enamel/composite border (**b**).

Sample 1 was selected as the material for the restoration of a tooth (filling up a cavity). The tooth was sectioned and the EDX mapping was performed on the dentin–resin interface and the enamel–resin interface (spectral accumulation was 15 min). The SEM imaging and mapping were focused on the microcracking at the interface. Figure 2a shows the fracture between the dentin/microcomposite. The fracture is large, probably due to the sectioning process. The EDX mapping shows the major components of the two materials: the dentin predominantly has P and Ca, while the composite has large amounts of Si, Al, and Ba. Other elements (Na, F, Mg, K, Cl, and N) are present in both dentin and composite in trace amounts, close to the limit of detection.

However, for the enamel/composite border (Figure 2b) a different situation is encountered. The composite shows large amounts of Si, Al, and Ba, but the enamel part has no distinct elements (on its own). Common elements (Ca, F, Mg, K, N, Na, *p*, Cl, and Zr) suggest that their concentration is similar for the two materials, both natural and restorative.

This means that the formulation for the microcomposite is similar to the natural formulation of the enamel. In the SEM micrographs it is evident that the border between the enamel and microcomposite is welded whereas the dentin/microcomposite border has an evident fracture. This fracture in the dentin/composite could also be due to the fact that we did not use Ag+ to dry the dentin prior to the experiment.

### 3.3. Raman Spectroscopy

**Figure 3:**
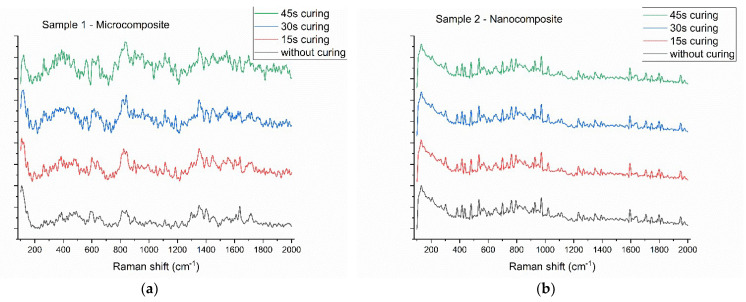
Raman spectra of the microcomposite (**a**) and nanocomposite (**b**) following different curing intervals.

Raman results (Figure 3) show a similar behavior for both samples 1 and 2, because both samples contain similar polymers. The main differences appear due to the filler: for the microcomposite (Figure 3a), bending of the ZrO bond appears at 1000 cm^−1^ [13] and at 806 cm^−1^ for the bending of the Si_2_O; in the case of the nanocomposite, there are low intensity peaks at 806 cm^−1^ and 1200 cm^−1^ (associated with the stretching of the SiO) [11]. Common peaks are associated with different vibrations of the carboxyl groups: at 1300 cm^−1^ (stretching of the C=O bond), 1404 cm^−1^ (stretching of the C=CH_2_), 1446 cm^−1^ (stretching of the aromatic ring), 1609 cm^−1^ (skeletal vibration of the aromatic ring), 1640 cm^−1^ (stretching of the C=C bond in methacrylate), and 1714 cm^−1^ (stretching of the C=O bond) [11]. Comparing the spectra from uncured to 45 s curing, we can observe variations regarding peak intensities, which suggest the presence of unreacted monomer in both samples. Furthermore, considering that the spectra were obtained from roughly the same spot, the spectra do not follow a monotonous trend because a rapid saturation is expected on the surface of the sample. Additionally, the variation of background is negligible comparing to the prominent Raman peaks.

From the Raman spectrum we can calculate the degree of conversion for the composites, using the following equation [11,13,23,24]:

(2) $$DC\left(\%\right)=\left|1-\frac{I{\left(1609\right)}_{45s}/I\;{\left(1640\right)}_{45s}}{I{\left(1609\right)}_{without\;curing}/I{\left(1640\right)}_{without\;curing}}\right|\times 100$$

Thus, for the two composites we obtain: 59% degree of conversion for Sample 1 and 93% for Sample 2, after 45 s of curing. These results could be correlated with the experimental data obtained via pycnometric measurements, but we have to consider that each technique has its own particularity: the pycnometry gives the value of the entire sample (by considering all the pores) whereas Raman spectroscopy measures the scattering of photons on imperfect surfaces. Thus, the surface electronic states influence the shape of the Raman peaks and are a “snapshot” of the irregular surfaces of the samples.

**Figure 4:**
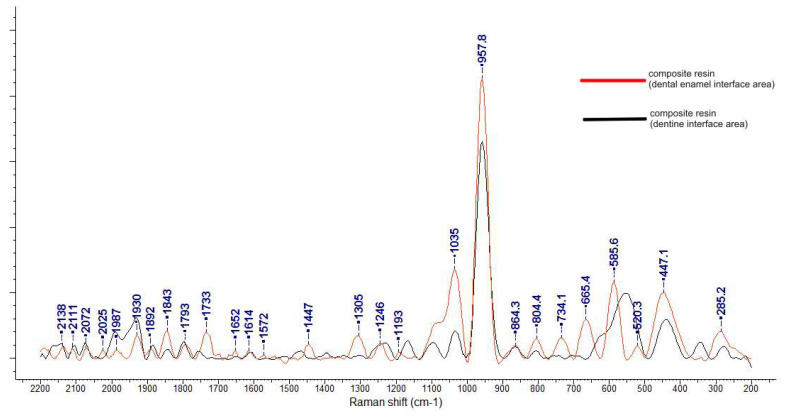
Raman spectra of the enamel-resin interface and dentin-resin interface.

For the restored tooth, the Raman spectra were obtained at the enamel–resin and dentin–resin interfaces and are shown in Figure 4. In this case, the Raman spectra are different than the Raman spectra of the composites: we cannot identify with precision the components (e.g., ZrO is obstructed, while Si_2_O and SiO have low intensity peaks) and the stretching and bending of the carboxyl and carbonyl groups are shifted. The skeletal vibration of the aromatic ring at 1609 cm^−1^ is shifted at 1624 cm^−1^, whereas the stretching of the methacrylate at 1640 cm^−1^ completely disappears, thus indicating a complete polymerization.

## 4. Conclusions

Two types of composites were studied and the degree of curing was assessed. The results from the pycnometric data seem to conclude that Sample 1 (microcomposite) has a degree of conversion higher than Sample 2 (nanocomposite). The SEM and EDX micrographs showed that Sample 1 has a porous surface while Sample 2 presents with a more compact surface, although not completely without holes. The elemental dispersion for the tooth restoration showed that a good bonding is obtained at the enamel–resin interface because of its predominant inorganic structure, while a new kind of bonding is required for the dentin–resin interface (the polymer is vulnerable at the dentin–composite interface). Both samples have the same variation in density during polymerization (~21%). The density difference comes from fillers, as Sample 1 contains ZrO_2_/SiO_2_ versus SiO_2_ only for Sample 2 (ZrO_2_ density is 5.68 g/cm^3^ while SiO_2_ density is 2.65 g/cm^3^). After calculating V_initial_–V_final_, we obtained a difference of 5.375 mm^3^ for Sample 1 and 4.375 mm^3^ for Sample 2. The larger increase of the volume for Sample 1 is related to a much larger porosity as proved by SEM images. This reasoning also explains the lower degree of conversion as obtained by Raman spectroscopy in the same geometry. This means that the same volume is probed for both samples, but Sample 1 is more porous, which means less amount of polymer is probed for Sample 1. Further investigations are necessary to assess the influence of water and nitrogen upon shrinkage and conversion rate.

## Figures and Tables

**Table 1:** Characteristics of the two samples investigated in the present study.

Properties/Samples	Sample 1 (Valux^TM^)	Sample 2 (Brilliant^TM^)
Manufacturer	3M ESPE (USA)	Coltène (Switzerland)
Composition	microcomposite	nanocomposite
Curing light	Visible light	Visible light
Curing time	40 s	20 s
Resin components	Bis-GMA and TEGDMA	TEGDMA, BIS-EMA
Inorganic filler type	zirconia/silica	Amorphous silica
Filler loading (vol.)	66%	65%
Particle size interval (μm)	0.01–3.5	0.02–2.5
Filler content by volume	66%	65 %

**Table 2:** Pycnometric results for the two investigated samples.

Properties/Samples	Sample 1 (Valux^TM^)	Sample 2 (Brilliant^TM^)
Density (kg/m^3^) before curing	1931.447	1560.784
Density (kg/m^3^) after curing	1514.511	1217.557
Variation (%)	21.58	21.99

## Data Availability

Not applicable.
